# Agreement, Calibration, and Exploratory Performance of AI-Based Ultrasound in Thyroid Nodule Assessment

**DOI:** 10.3390/jcm15145323

**Published:** 2026-07-08

**Authors:** Dorota Szydlarska, Marta Ciechomska, Karolina Kędzierska-Kapuza, Edward Franek, Katarzyna Dźwiarek-Miara, Magdalena Łukawska-Tatarczuk, Iwona Kaczor-Zabój

**Affiliations:** 1 Department of Digital Medicine, Implementation and Innovation, National Medical Institute of the Ministry of the Interior and Administration, 02-507 Warsaw, Poland; 2 Department of Family Medicine, Medical University of Warsaw, 02-091 Warsaw, Poland; 3 Family Medicine Clinic, National Medical Institute of the Ministry of the Interior and Administration, 02-507 Warsaw, Poland; marta.ciechomska@pimmswia.gov.pl; 4 Diabetology Center, National Medical Institute of the Ministry of the Interior and Administration, 02-507 Warsaw, Poland; 5 Department of Internal Medicine, Endocrinology and Diabetology, National Medical Institute of the Ministry of the Interior and Administration, 02-507 Warsaw, Polandmagdalena.lukawska@pimmswia.gov.pl (M.Ł.-T.);

**Keywords:** thyroid nodule, artificial intelligence, ultrasound, medical imaging

## Abstract

**Background/Objective:** Ultrasound is the first-line imaging modality for thyroid nodule assessment; however, it remains highly operator-dependent and subject to interobserver variability. Artificial intelligence (AI)-based systems have been proposed to improve reproducibility, yet evidence regarding their agreement with clinician assessment—particularly at the level of individual sonographic features—remains limited. Importantly, most available studies evaluate concordance rather than true diagnostic accuracy against an independent reference standard. To evaluate agreement between an AI-based ultrasound system and expert clinician assessment in thyroid nodule evaluation, focusing on concordance of size measurements, agreement in sonographic feature classification, and exploratory diagnostic performance relative to cytological outcomes. **Methods:** This retrospective single-center study included 74 thyroid nodules from adult patients undergoing routine ultrasound examination. Archived ultrasound images were independently assessed by an experienced clinician and an AI-based system. Agreement for quantitative measurements was evaluated using Bland–Altman analysis, while categorical features were assessed using percent agreement and Cohen’s kappa coefficients. Calibration was examined using scatter plots with the line of identity. Cytological results, when available, were used as a non-uniform exploratory reference standard for diagnostic analyses. Exploratory diagnostic performance was assessed using receiver operating characteristic (ROC) curves and area under the curve (AUROC) estimates. Given the study design, analyses primarily reflect agreement and measurement concordance rather than true diagnostic accuracy. **Results:** AI-derived and clinician measurements demonstrated strong agreement across all dimensions, with minimal systematic bias and stable calibration patterns. A small but consistent underestimation of one measurement axis by approximately 1 mm was observed. For categorical features, agreement ranged from fair to moderate (κ = 0.196–0.368), with the highest concordance for echogenic foci and lowest for echogenicity. Exploratory analyses showed variable diagnostic discrimination, with the best performance observed for size measurements and selected sonographic features. **Conclusions:** AI-based ultrasound analysis demonstrates robust agreement with clinician assessment for quantitative thyroid nodule measurements, while agreement for categorical feature classification remains moderate and variable. The findings highlight that the present study evaluates concordance rather than definitive diagnostic accuracy, particularly given the lack of a uniform independent reference standard. These results support the role of AI as an assistive tool in thyroid ultrasound practice, improving measurement reproducibility while requiring ongoing clinician oversight for qualitative interpretation.

## 1. Introduction

Thyroid nodules represent one of the most common endocrine findings in clinical practice, with a prevalence ranging from 19% to 68% when detected by high-resolution ultrasound (US), although only a small proportion prove to be malignant. Ultrasound has therefore become the first-line imaging modality for thyroid nodule assessment owing to its wide availability, non-invasiveness, and cost-effectiveness. However, despite the use of standardized reporting systems, ultrasound evaluation remains highly operator-dependent and is subject to considerable interobserver variability, particularly for features such as echogenicity, margins, and echogenic foci [1,2]. To address this variability and improve risk stratification, several ultrasound-based classification systems have been developed, including ACR TI-RADS, EU-TIRADS, ATA guidelines, and other national frameworks. These systems rely on visual interpretation of predefined sonographic features—composition, echogenicity, shape, margins, and calcifications—that are weighted to estimate malignancy risk and guide indications for fine-needle aspiration (FNA). Although these systems enhance standardization, multiple studies have demonstrated only fair-to-moderate agreement for several individual features, even among experienced endocrinologists, highlighting intrinsic limitations of human visual assessment [2,3]. In recent years, artificial intelligence (AI)-based solutions, particularly computer-aided diagnosis (CAD) and deep-learning systems, have emerged as promising tools in thyroid ultrasound. AI systems can automatically extract and analyze image features, offering objective and reproducible assessments that may reduce interobserver variability and support clinical decision-making. Multiple studies have reported high agreement and discrimination analysis of AI-assisted ultrasound for differentiating benign and malignant thyroid nodules. Moreover, AI systems have demonstrated substantial time-efficiency advantages, suggesting a potential role in high-volume clinical workflows [4,5]. Despite these encouraging results, important limitations remain. Many AI studies focus primarily on exploratory performance metrics, such as sensitivity, specificity, or the area under the receiver operating characteristic curve (AUC), while comparatively less attention has been paid to feature-level agreement between AI systems and clinicians. Furthermore, several high-performance AI models function as “black boxes,” which may limit clinical trust and hinder implementation in routine endocrine practice. Recent investigations have therefore emphasized the need for transparent validation studies that directly compare AI-based feature assessment with clinician interpretation using established agreement measures, such as Cohen’s kappa coefficient [6,7]. Additionally, while AI has shown promising accuracy for malignancy prediction, evidence regarding its ability to reliably predict cytological outcomes—particularly indeterminate categories—based on individual ultrasound features remains inconsistent. This underscores the need for cautious evaluation of AI as a supportive rather than standalone diagnostic tool, especially in clinical settings where cytology remains the standard of care [8,9].

Against this background, the present study aimed to compare an AI-based ultrasound system with experienced endocrinologists assessment in the evaluation of thyroid nodules, focusing on: (1) agreement for individual sonographic features, (2) concordance of size measurements, and (3) exploratory diagnostic discrimination using cytology as the reference standard. By prioritizing agreement analyses alongside clinically interpretable outcomes, this study aims to delineate the practical role of AI as a supportive tool in routine thyroid ultrasound rather than a replacement for human assessment.

### Objectives

The aim of this study was to evaluate the performance of an AI-based ultrasound system in the assessment of thyroid nodules in comparison with experienced endocrinologists interpretation. Specifically, the study aimed to:

(1) assess the agreement between the AI system and experienced endocrinologists for individual sonographic features,
(2) evaluate the concordance of nodule size measurements, and
(3) explore the agreement and discrimination analysis of the AI system in differentiating benign and malignant nodules using cytology as the reference standard.

## 2. Material

### 2.1. Study Design and Population

This single-center retrospective study was conducted at the Endocrinology Outpatient Clinic of the National Medical Institute of the Ministry of the Interior and Administration in Warsaw, Poland. We analyzed data from adult patients who underwent routine thyroid ultrasonography within a predefined study period and had complete archived ultrasound documentation, enabling parallel evaluation by an experienced clinician and an artificial intelligence (AI)-based system. A total of 74 thyroid nodules were included in the analysis. Detailed characteristics of the study population, including age, sex, nodule location, and maximum nodule diameter, are presented in Table 1.

Exclusion criteria included incomplete ultrasonographic documentation, insufficient image quality precluding AI analysis, and the absence of reference data required for comparative evaluation.

### 2.2. Ultrasound Examination and AI Analysis

All ultrasound examinations were performed as part of routine outpatient clinical practice by an experienced clinician. Subsequently, the same archived ultrasound images were analyzed using a commercially available AI system developed for thyroid nodule assessment. The analysis included measurements of nodule dimensions (transverse, anteroposterior, and longitudinal) as well as evaluation of morphological features in accordance with a standardized ultrasonographic lexicon, including nodule composition, echogenicity, shape, margins, and the presence of echogenic foci. All examinations were performed using a Mindray Resona I9 ultrasound system equipped with a high-frequency linear transducer (L14–3Ws). Ultrasound studies were conducted according to a uniform clinical protocol, with comparable machine settings applied across examinations to minimize the impact of technical variability on image acquisition.

### 2.3. Ethical Considerations

The study protocol was approved by the local Bioethics Committee of the National Medical Institute of the Ministry of the Interior and Administration in Warsaw (approval number: 29/2026). Owing to the retrospective design of the study, the use of data obtained during routine clinical diagnostics, and the absence of any intervention in the diagnostic or therapeutic process, the requirement for individual written informed consent was waived by the Bioethics Committee. The study was conducted in accordance with the principles of the Declaration of Helsinki.

## 3. Methods

The AI analysis was performed using a commercially available software system Mindray Resona I9 (Shenzhen Mindray Bio-Medical Electronics Co., Ltd., Shenzhen, China). The system is designed for automated assessment of thyroid nodules based on ultrasound images, including feature extraction and classification according to a standardized ultrasound lexicon. The software operates as a proprietary system, and detailed information regarding model architecture and training data is not publicly available. During the study period, the system was used in a locked mode, without adaptive updates. AI analysis was performed without manual region-of-interest selection, based on a single representative image per nodule. All included cases successfully underwent AI analysis; examinations with insufficient image quality were excluded prior to inclusion in the study. All statistical analyses were performed using Python (version 3.12) with standard scientific libraries. Categorical features were harmonized to a common ultrasound lexicon based on predefined mapping rules aligning AI outputs with ACR TI-RADS terminology.

Fine-needle aspiration cytology (FNAC) served as the reference standard for exploratory diagnostic analyses. However, the primary aim of the study was to assess agreement between AI-based assessment and clinician evaluation.

Agreement between AI-based and experienced endocrinologist-based measurements of thyroid nodule dimensions was assessed using Bland–Altman analysis. For each transverse, anteroposterior, and longitudinal diameter, the mean of the paired measurements and their differences were calculated. The mean difference (bias) and the 95% limits of agreement were estimated and visualized using Bland–Altman plots. Measurement calibration was further evaluated using scatter plots comparing AI-derived and experienced endocrinologists measurements across the observed size range, with the line of identity (y = x) included to assess proportional bias and consistency. Interpretation focused on the clinical acceptability of agreement and visual assessment of concordance rather than formal hypothesis testing.

Cytological data were available for a subset of nodules and were used for exploratory analyses only. All ultrasound examinations and reference assessments were performed by four endocrinologists with more than 5 years of experience in thyroid ultrasound. Examinations were conducted as part of routine clinical practice using a standardized acquisition protocol. The term “manual reference” refers to ultrasound assessment performed by an experienced endocrinologist during routine clinical examination. Human assessments were performed independently and were not influenced by AI outputs or cytological results at the time of ultrasound examination. AI analysis was conducted retrospectively on archived images. Due to the retrospective design and reliance on routine clinical assessments, formal intra-reader and inter-reader variability were not evaluated.

AI measurements were treated as continuous scores for receiver operating characteristic (ROC) analysis, with area under the ROC curve (AUROC) estimated using trapezoidal integration and Youden-optimal operating points identified. For composition, echogenicity, shape, margin, and echogenic foci, variables were binary or recoded to binary, with solid composition, hypoechogenicity, and the presence of echogenic foci (any nonzero entry) defined as positive. AI-based size measurements proved clinically informative, with the longitudinal diameter demonstrating the strongest discriminatory performance among the evaluated size pairs while maintaining high sensitivity and specificity at the Youden-optimal threshold. Hypoechogenicity was robustly identified by the AI system, showing a well-balanced and high sensitivity and specificity profile. Echogenic foci were detected with high sensitivity and good overall discriminatory ability. In contrast, composition and shape tended to prioritize sensitivity over specificity, whereas margin assessment showed a more balanced performance. These findings suggest that greater feature subtyping—such as differentiation between punctate and macro- or rim-type calcifications—and human adjudication may help reduce false-positive classifications. Methodological considerations included the use of Wilson 95% confidence intervals for proportions, nonparametric bootstrap 95% confidence intervals for AUROC estimates, and DeLong tests for pairwise comparison of AUROCs across size measurements drawn from a shared case set. Sensitivity analyses demonstrated that the principal clinical conclusions were robust across a range of ground-truth thresholds (5–15 mm), with the longitudinal diameter consistently emerging as the best-performing axis.

Archived ultrasound datasets included both static images and cine loops. However, Doppler imaging and elastography were not systematically available and were not included in the analysis. For each nodule, the AI system analyzed a single representative image selected from the archived examination.

## 4. Results

### 4.1. Study Cohort

A total of 74 thyroid nodules were included in the final analysis. Mean age was 62.3 ± 15.7 years (median 65.0, IQR 47.8–73.0), and most nodules were observed in female patients (67/74, 90.5%). Nodules were distributed between the right (50.0%) and left (47.3%) thyroid lobes, with 4.1% located in the isthmus. The mean largest nodule diameter was 17.3 ± 9.9 mm (Table 1).

### 4.2. Agreement and Calibration of Size Measurements

**Figure 1:**
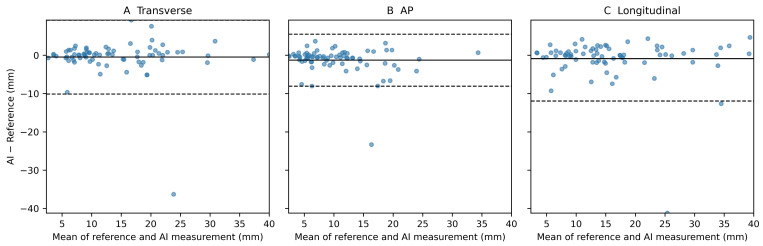
Bland–Altman analyses of measurement agreement. (**A**) Transverse dimension. (**B**) Anteroposterior (AP) dimension. (**C**) Longitudinal dimension.

**Figure 2:**
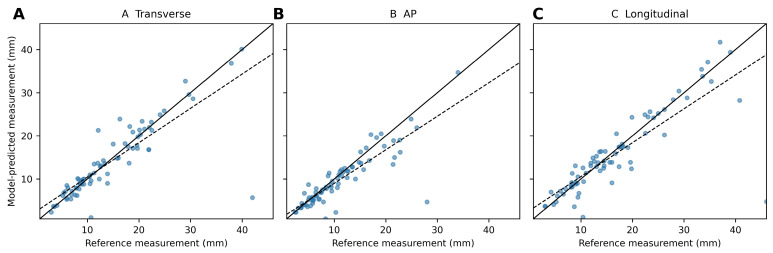
Calibration of model-predicted measurements. (**A**) Transverse dimension. (**B**) Anteroposterior (AP) dimension. (**C**) Longitudinal dimension.

**Figure 3:**
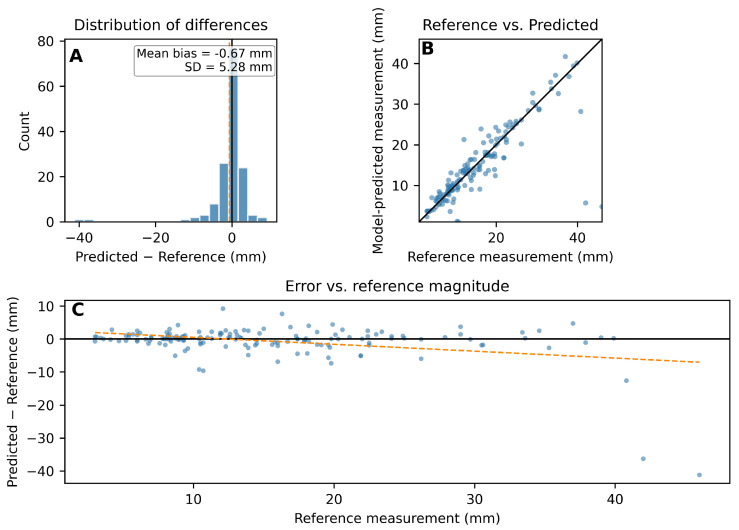
Global error characteristics of model-predicted measurements. (**A**) Distribution of differences between reference and model-predicted values across the study cohort, illustrating overall bias and dispersion. (**B**) Scatter plot showing the relationship between reference and model-predicted measurements, with the line of identity. (**C**) Prediction error as a function of reference measurement magnitude with a linear trend line to assess scale-dependent behavior. Prediction error as a function of reference measurement magnitude. The dotted line represents the fitted linear trend used to assess scale-dependent behavior.

Bland–Altman analyses demonstrated close agreement between AI-derived and reference measurements across all three dimensions (Figure 1A–C). Transverse and longitudinal diameters showed minimal systematic bias, whereas a small, consistent underestimation of the anteroposterior (AP) diameter by the AI system was observed, on the order of approximately 1 mm (Figure 1B). Calibration plots confirmed a strong linear relationship between AI-predicted and reference measurements for all axes, closely following the line of identity across the observed size range (Figure 2). Global error analyses showed symmetric error distributions without clear scale-dependent trends, indicating stable calibration and absence of proportional bias (Figure 3). Intraclass correlation coefficients (ICC) exceeded 0.80 for all measured dimensions, indicating strong agreement between AI and human measurements.

### 4.3. Inter-Method Agreement for Categorical Ultrasound Features

**Figure 4:**
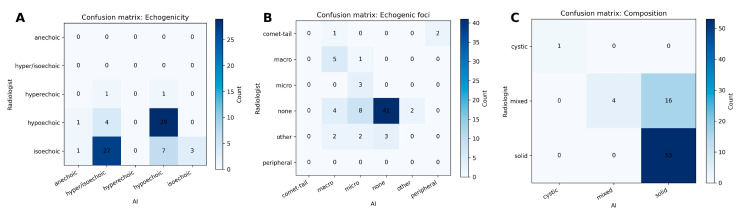
Confusion matrices illustrating classification agreement for echogenicity, echogenic foci, and composition. Confusion matrix illustrating classification performance for echogenic foci using normalized terminology. Rows correspond to reference labels and columns to predicted labels. Color intensity reflects the normalized frequency of observations. (**A**) Echogenicity. (**B**) Echogenic foci. (**C**) Composition.

After harmonization to a common ultrasound lexicon, agreement between AI and experienced endocrinologists assessments of categorical features was fair to moderate. Percent agreement ranged from 43.2% for dominant echogenicity to 81.1% for shape, with Cohen’s κ values between 0.196 and 0.368 across evaluated features (Table 2). Agreement was highest for echogenic foci, whereas dominant echogenicity showed the lowest concordance. Confusion-matrix analysis for echogenic foci illustrated that most disagreements reflected adjacent or overlapping categories rather than systematic misclassification, supporting partial concordance after terminology normalization (Figure 4).

### 4.4. Exploratory Discrimination of AI Outputs Against Manual Reference

In exploratory analyses, AI-based size measurements demonstrated variable discrimination when benchmarked against manually defined size thresholds. Among the evaluated axes, the longitudinal diameter showed the most consistent performance across ground-truth cut-offs, whereas transverse and AP measurements exhibited greater variability (Table 3; Supplementary Tables S1a and S2). For categorical features, discrimination against manual reference was strongest for dominant echogenicity and echogenic foci, while composition and shape tended to favor sensitivity at the expense of specificity (Table 3; Supplementary Table S1b). Given the reliance on manual ground truth, these analyses were interpreted as supportive rather than confirmatory and were therefore considered secondary to agreement and calibration outcomes.

## 5. Discussion

Reported AI performance varies widely across datasets, acquisition protocols, and disease prevalence. Many published studies rely on retrospective, single-center cohorts, while head-to-head comparisons with human readers often lack standardized endpoints or rigorous prospective designs.

To address these limitations, international reporting frameworks—CONSORT-AI and SPIRIT-AI for clinical trials of AI interventions, and the recently introduced STARD-AI for exploratory performance studies involving AI—provide structured guidance to improve transparency in dataset curation, human–AI interaction, error analysis, algorithm versioning, and bias and fairness considerations. Adherence to these frameworks is increasingly recognized as essential for generating interpretable and generalizable evidence capable of informing regulatory evaluation and clinical adoption [10,11,12].

This study has several limitations. First, it is based on a relatively small, single-center dataset consisting of 74 thyroid nodules. Such a limited sample size may increase the risk of overestimating agreement measures and reduce the robustness of the findings. Additionally, the single-center design may limit the generalizability of the results to other populations and clinical settings. Therefore, the findings should be interpreted with caution and considered as exploratory. Future multicenter studies with larger cohorts are needed to validate these results.

The relatively small sample size and limited availability of comprehensive cytological or histopathological reference data further restrict the ability to draw definitive conclusions regarding exploratory performance. Cytological data were used for exploratory purposes and not as a definitive diagnostic reference for all cases.

Limited transparency regarding the internal functioning of the proprietary AI system, including training data and algorithm architecture, may affect interpretability and external validity.

Against this background, the present study demonstrates high agreement between AI-based and manual endocrinologist measurements of thyroid nodule dimensions, with ICC (2,1) values exceeding 0.80 across all axes. A small but statistically significant underestimation of the anteroposterior (AP) diameter by AI—on the order of approximately 1 mm—was consistently identified using Bland–Altman and calibration analyses. In contrast, no significant differences were observed for transverse or longitudinal measurements after correction for multiple testing. These results support the use of AI as a reliable assistant for quantitative nodule sizing, while underscoring that even modest, axis-specific systematic deviations merit attention when clinical decisions hinge on size-based thresholds, such as TI-RADS biopsy cut-offs. This interpretation is concordant with prior work showing that deep-learning systems can match experienced endocrinologists performance in thyroid nodule risk stratification and may enhance measurement standardization when terminology is harmonized and models are appropriately calibrated [13,14].

Calibration analyses further revealed an approximately linear relationship between AI-derived and manual measurements across the observed size range, closely tracking the line of perfect agreement. Importantly, this calibration pattern remained qualitatively stable in stratified analyses by sex, thyroid lobe, and nodule size category, suggesting consistent algorithm behavior across clinically relevant subgroups. Nonetheless, limited sample sizes in the smallest and largest nodules constrain the precision of these subgroup estimates and preclude definitive conclusions at the extremes of the size spectrum.

For categorical ultrasound features, inter-method agreement was limited overall, with Cohen’s kappa values indicating only slight-to-fair to fair agreement depending on the feature. Although percent agreement was relatively high for some features (e.g., shape), the corresponding kappa values were low, likely reflecting a prevalence effect and limited agreement beyond chance. These findings underscore that percent agreement alone may overestimate true concordance and that categorical interpretation remains challenging for AI systems. In particular, agreement for clinically important TI-RADS features such as echogenicity, margins, and shape was suboptimal, which may limit direct clinical applicability. While normalization to a standardized ultrasound lexicon improved comparability between AI and human assessments, the overall level of agreement remained modest. This suggests that standardization alone is insufficient and that further methodological refinement is required before categorical AI outputs can be reliably integrated into risk stratification frameworks.

These results align with the broader literature. A recent meta-analysis reported pooled AI sensitivity of 0.86 and specificity of 0.78 (AUC 0.89), not inferior to r experienced endocrinologists (AUC 0.91) [13]. In a prospective real-world study, AI achieved agreement and discrimination analysis comparable to that of senior experienced endocrinologists and significantly improved junior readers’ accuracy, particularly for nodules ≤ 1.5 cm, while reducing potentially unnecessary biopsies in larger nodules—an effect with direct implications for clinical workflow and patient management [14]. Independent validation studies across ultrasound vendors further demonstrate that device-specific differences influence absolute performance for both AI systems and human readers, highlighting the importance of external validation and ongoing vigilance for domain shift [15].

Technical reviews describe a maturing AI pipeline evolving from detection and segmentation to comprehensive classification, with segmentation-based approaches—such as U-Net, TransUNet, or CNN–ViT hybrids—supporting more reproducible dimensional measurements that are directly relevant to TI-RADS decision thresholds [16,17]. Implementation-focused analyses further emphasize that FDA-cleared products vary in how they integrate with TI-RADS workflows and stress the necessity of careful deployment, continuous performance monitoring, and strict adherence to ACR lexicon standards [18]. Global epidemiological analyses from GLOBOCAN highlight a high incidence but low mortality of thyroid cancer, with this divergence largely attributed to overdiagnosis [19,20]. Within this context, AI systems should aim not merely to maximize sensitivity, but to help rebalance sensitivity and specificity in a manner that reduces unnecessary procedures without compromising oncological safety.

Analysis of measurement discrepancies suggests that larger differences between AI and human assessments may arise in more challenging imaging conditions. Based on visual inspection of outlier cases in Bland–Altman plots, discrepancies were most frequently associated with heterogeneous nodules, partially cystic composition, indistinct or irregular margins, and reduced image quality. Additional contributing factors may include posterior acoustic shadowing and difficulties in defining precise lesion boundaries, particularly in nodules with complex internal architecture. These observations highlight typical scenarios in which AI-based segmentation and measurement may be less reliable and reinforce the importance of maintaining clinician oversight, especially in technically challenging cases. Future studies incorporating systematic segmentation-level evaluation and representative imaging examples would be valuable for a more detailed characterization of AI error patterns.

### Clinical Implications

(1) Quantitative measurements: The high ICC values support incorporating AI into routine nodule sizing, with potential benefits in examination efficiency and reproducibility—particularly where maximum diameter determines TI-RADS-based recommendations for FNA or follow-up. The modest AP bias (~1 mm) suggests that simple calibration offsets or context-aware alerts at decision thresholds could mitigate clinically relevant effects [21].
(2) Qualitative features: After normalization to the ACR TI-RADS lexicon, agreement improves for features such as echogenic foci, supporting the implementation of explicit terminology mapping (e.g., punctate echogenic foci to microcalcifications) within AI user interfaces and structured reporting templates [21].
(3) Deployment and oversight: Real-world evidence indicates susceptibility to vendor- and device-related domain shifts, underscoring the need for local validation before full clinical deployment and for continuous quality assurance of both measurements and categorical classifications [15,18].

Importantly, thyroid nodule management is based on integrated risk stratification rather than isolated ultrasound features. Therefore, discrepancies observed between AI and human assessment at the feature level—particularly for echogenicity and margins—may have direct clinical consequences through their influence on TI-RADS scoring. Even small differences in feature classification can lead to changes in TI-RADS category assignment, potentially affecting recommendations for fine-needle aspiration (FNA), surveillance intervals, and patient counseling.

In this context, the fair-to-moderate agreement observed for selected categorical features should be interpreted cautiously. Misclassification by AI may theoretically result in both over-biopsy, due to overestimation of suspicious characteristics, and under-biopsy, due to failure to detect high-risk features. These findings reinforce that AI systems should currently be regarded as supportive tools rather than standalone decision-making systems, particularly in cases where management decisions depend on subtle qualitative features.

Our results further underscore the importance of maintaining clinician oversight and integrating AI outputs within a broader clinical framework that includes patient risk factors, longitudinal follow-up, and cytological assessment where indicated. While the present study was not powered for definitive analysis of downstream clinical decisions, these considerations highlight the need for future research evaluating the impact of AI-assisted ultrasound on biopsy rates, follow-up strategies, and patient outcomes, ideally with stratification according to Bethesda categories.

Key strengths of this study include paired AI–human comparisons on identical cases and the combined use of agreement, calibration, and discriminatory analyses, extending beyond classification metrics alone. Limitations include potential domain shift related to probe type, acquisition settings, and disease prevalence, which may affect generalizability. In addition, the lack of external validation limits the generalizability of the findings across different clinical settings and ultrasound systems.

Additionally, small sample sizes in extreme nodule-size strata limit estimate precision and regression stability, as noted in prior prospective and implementation studies [14,15,18].

Given the persistent heterogeneity of study designs and under-specified human–AI interactions in this field, rigorous application of CONSORT-AI and SPIRIT-AI for interventional studies and STARD-AI for exploratory performance research remains essential. Explicit reporting of algorithm versioning, input data handling, external validation procedures, error and bias analyses, and fairness considerations will be critical to enable assessment of quality, transferability, and clinical readiness by both readers and regulators [10,11,12].

Future research should prioritize prospective, multicentre investigations with external validation across ultrasound platforms, reported in accordance with STARD-AI. Such studies should quantify downstream clinical impact—including changes in biopsy rates, reporting time, cost-effectiveness, and equity-aware performance—and incorporate TI-RADS-aligned deployment strategies with adaptive calibration and active performance surveillance [12,18].

Additionally, the absence of formal intra- and inter-reader variability assessment limits conclusions regarding reproducibility across different observers.

## 6. Conclusions

To conclude, our results indicate that AI-based ultrasound analysis demonstrates robust agreement with manual assessment for quantitative measurements, while categorical feature classification remains limited and requires further refinement.

Given the exploratory design and limited sample size, these findings should be interpreted with caution. The study supports the potential role of AI as a supportive tool in thyroid ultrasound, but further large-scale, prospectively validated studies with comprehensive cytological or histopathological reference are required before clinical implementation.

Highlights:

1. AI shows strong agreement with human assessment for quantitative thyroid nodule measurements.
2. Categorical feature classification remains limited and requires further refinement despite the use of standardized terminology.
3. Study generalizability may be affected by imaging protocols, probe types, and disease prevalence.
4. Adherence to CONSORT-AI, SPIRIT-AI, and STARD-AI ensures transparency, reproducibility, and clinical applicability.
5. Integration of AI into routine thyroid ultrasound is supported by stable calibration and modest, predictable bias, provided human oversight is maintained.

## Figures and Tables

**Table 1:** Baseline characteristics of thyroid nodules included in the study. Demographic characteristics and nodule distribution for the analyzed cohort. Age is reported as mean ± standard deviation (SD) and median with interquartile range (IQR). Nodule characteristics include patient sex, laterality (right/left), isthmus location, and largest nodule diameter (mm, mean ± SD). Abbreviations: SD, standard deviation; IQR, interquartile range.

Variable	Value
N nodules	74
Age (years), mean ± SD	62.3 ± 15.7
Age (years), median (IQR)	65.0 (47.8–73.0)
Female, *n* (%)	67 (90.5%)
Location: right, *n* (%)	37 (50.0%)
Location: left, *n* (%)	35 (47.3%)
Location: isthmus, *n* (%)	3 (4.1%)
Largest diameter (mm), mean ± SD	17.3 ± 9.9

**Table 2:** Inter-method agreement between AI and experienced endocrinologists for categorical ultrasound features (Cohen’s κ). Percent agreement and Cohen’s kappa (κ) between AI and experienced endocrinologists assessments for composition, echogenicity, shape, margin, and echogenic foci. Features were normalized to a common lexicon prior to analysis. κ values indicate fair-to-moderate inter-method agreement across most categorical ultrasound features.

Feature	Percent Agreement	Cohen’s Kappa
Composition	78.4	0.319
Echogenicity	43.2	0.243
Shape (wider-than-tall)	81.1	0.196
Margin (smooth)	71.8	0.314
Echogenic foci	66.2	0.368

**Table 3:** Agreement and discrimination analysis summary (AUROC with 95% confidence intervals) across clinical practitioner–AI feature pairs.

Clinical Practitioner	AI	AUROC	AUROC 95% CI (Low)	AUROC 95% CI (High)
transverse diameter (mm)	AI transverse diameter (mm)	0.613	0.449	0.802
AP diameter (mm)	AI AP diameter (mm)	0.608	0.440	0.761
longitudinal diameter (mm)	AI longitudinal diameter (mm)	0.797	0.431	0.855
composition	AI composition	0.603	0.536	0.682
dominant echogenicity	AI echogenicity	0.839	0.752	0.918
shape	AI shape	0.585	0.455	0.720
margin	AI margin	0.775	0.631	0.871
echogenic foci	AI echogenicity foci	0.796	0.690	0.892

## Data Availability

The original contributions presented in this study are included in the article/Supplementary Material. Further inquiries can be directed to the corresponding author.

## Supplementary Materials

The following supporting information can be downloaded at: https://www.mdpi.com/article/10.3390/jcm15145323/s1, Supplementary Table S1. Operating-point performance at Youden-optimal thresholds with confusion-matrix counts; Supplementary Table S2. Sensitivity analysis across ground-truth thresholds for AI size measurements.

## References

[1] Hoang J.K., Middleton W.D., Farjat A.E., Teefey S.A., Abinanti N., Boschini F.J., Bronner A.J., Dahiya N., Hertzberg B.S., Newman J.R. (2018). Interobserver variability of sonographic features used in the American College of Radiology Thyroid Imaging Reporting and Data System. AJR Am. J. Roentgenol..

[2] Persichetti A., Di Stasio E., Coccaro C., Graziano F.M., Bianchini A., Di Donna V., Corsello S.M., Valle D., Bizzarri G., Frasoldati A. (2020). Inter- and intraobserver agreement in the assessment of thyroid nodule ultrasound features and classification systems. Thyroid.

[3] de Carlos J., Garcia J., Basterra F.J., Pineda J.J., Ollero M.D., Toni M., Munarriz P., Anda E. (2024). Interobserver variability in thyroid ultrasound. Endocrine.

[4] Xu C., Wang Z., Zhou J., Hu F., Wang Y., Xu Z., Cai Y. (2025). Application of artificial intelligence software in the analysis of thyroid nodule ultrasound image characteristics. PLoS ONE.

[5] Cai X., Zhou Y., Ren J., Wei J., Lu S., Gu H., Xu W., Zhu X. (2025). Intelligent diagnosis of thyroid nodules with AI ultrasound assistance and cytology classification. Front. Endocrinol..

[6] Cao C.-L., Li Q.-L., Tong J., Shi L.-N., Li W.-X., Xu Y., Cheng J., Du T.-T., Li J., Cui X.-W. (2023). Artificial intelligence in thyroid ultrasound. Front. Oncol..

[7] Chen Z., Chambara N., Liu S.Y.W., Chow T.C.M., Lai C.M.S., Ying M.T.C. (2025). Intra- and interobserver reliability of AI in thyroid nodule ultrasound feature analysis. Diagnostics.

[8] Madeo B., Brigante G., Ansaloni A., Taliani E., Kaleci S., Monzani M.L., Simoni M., Rochira V. (2020). The added value of operator’s judgement in thyroid nodule ultrasound classification arising from histologically based comparison of different risk stratification systems. Front. Endocrinol..

[9] Zhou T., Xu L., Shi J., Zhang Y., Lin X., Wang Y., Hu T., Xu R., Xie L., Sun L. (2024). US of thyroid nodules: Can AI-assisted diagnostic system compete with fine needle aspiration?. Eur. Radiol..

[10] Liu X., Rivera S.C., Moher D., Calvert M.J., Denniston A.K. (2020). CONSORT-AI Reporting guidelines for clinical trials evaluating artificial intelligence interventions: CONSORT-AI. BMJ.

[11] Cruz Rivera S., Liu X., Chan A.W., Denniston A.K., Calvert M.J. (2020). SPIRIT-AI Guidelines for clinical trial protocols for interventions involving artificial intelligence: SPIRIT-AI. Lancet Digit. Health.

[12] Sounderajah V., Guni A., Liu X., Collins G.S., Karthikesalingam A., Markar S.R., Golub R.M., Denniston A.K., Shetty S., Moher D. (2025). STARD-AI Reporting guidelines for diagnostic accuracy studies involving artificial intelligence: STARD-AI. Nat. Med..

[13] Potipimpanon P., Charakorn N., Hirunwiwatkul P. (2022). A comparison of artificial intelligence vs radiologists in thyroid ultrasound: A systematic review and meta-analysis. Eur. Arch. Otorhinolaryngol..

[14] Li Y., Liu Y., Xiao J., Yan L., Yang Z., Li X., Zhang M., Luo Y. (2023). Clinical value of artificial intelligence in thyroid ultrasound: A prospective real-world study. Eur. Radiol..

[15] Weng J., Wildman-Tobriner B., Buda M., Yang J., Ho L.M., Allen B.C., Ehieli W.L., Miller C.M., Zhang J., Mazurowski M.A. (2023). Deep learning for classification of thyroid nodules on ultrasound: Validation on an independent dataset. Clin. Imaging.

[16] Das D., Iyengar M.S., Majdi M.S., Rodriguez J.J., Alsayed M. (2024). Deep learning for thyroid nodule examination: A technical review. Artif. Intell. Rev..

[17] Savelonas M. (2025). An Overview of AI-Guided Thyroid Ultrasound Image Segmentation and Classification for Nodule Assessment. Big Data Cogn. Comput..

[18] Wildman-Tobriner B., Taghi-Zadeh E., Mazurowski M.A. (2022). AI tools for thyroid nodules on ultrasound. AJR Am. J. Roentgenol..

[19] Pizzato M., Li M., Vignat J., Laversanne M., Singh D., La Vecchia C., Vaccarella S. (2022). The epidemiological landscape of thyroid cancer worldwide (GLOBOCAN 2020). Lancet Diabetes Endocrinol..

[20] International Agency for Research on Cancer, World Health Organization (2022). GLOBOCAN 2022: Thyroid Cancer Fact Sheet.

[21] Tessler F.N., Middleton W.D., Grant E.G., Hoang J.K., Berland L.L., Teefey S.A., Cronan J.J., Beland M.D., Desser T.S., Frates M.C. (2017). ACR Thyroid Imaging Reporting and Data System (TI-RADS): White paper of the ACR TI-RADS Committee. J. Am. Coll. Radiol..

