# Supplementary material for: Agreement, Calibration, and Exploratory Performance of AI-Based Ultrasound in Thyroid Nodule Assessment
Source: J Clin Med. 2026 Jul 8;15(14):5323. doi: 10.3390/jcm15145323 (PMC13411397; doi:10.3390/jcm15145323)
Supplement: Supplementary file 1 [file jcm-15-05323-s001.zip › jcm-4389700-supplementary.pdf]

Supplementary Table S1. Operating-point performance at Youden-optimal thresholds with confusion-matrix counts

Sensitivity and specificity at Youden-optimal operating points for each manual–AI feature pair. Values are reported with Wilson 95% confidence intervals, along with AI decision thresholds and confusion-matrix counts (true positives, true negatives, false positives, and false negatives).

Table S1a. Operating-point performance (Youden-optimal) — size measurements

clinical practitioner	**AI**	**n**	**Se**	**Se 95% CI**	**Sp**	**Sp 95% CI**	**AI threshold**	**TP**	**TN**	**FP**	**FN**
transverse diameter (mm)	AI transverse diameter (mm)	74	0.870	0.743–0.939	1.000	0.879–1.000	10.500	40	28	0	6
AP diameter (mm)	AI AP diameter (mm)	74	0.914	0.776–0.970	0.923	0.797–0.973	8.900	32	36	3	3
longitudinal diameter (mm)	AI longitudinal diameter (mm)	74	0.923	0.818–0.970	0.955	0.782–0.992	11.400	48	21	1	4

Abbreviations: Se, sensitivity; Sp, specificity; AP, anteroposterior; GT, ground truth (manual). Sensitivity and specificity CIs are Wilson 95% CIs. Operating point defined by Youden's J. TP, true positive; TN, true negative; FP, false positive; FN, false negative.

Table S1b. Operating-point performance (Youden-optimal) — categorical features

clinical practitioner	**AI**	**n**	**Se**	**Se 95% CI**	**Sp**	**Sp 95% CI**	**AI threshold**	**TP**	**TN**	**FP**	**FN**
Composition	AI composition	74	1.000	0.923–1.000	0.207	0.098–0.384	—	46	6	23	0
dominant echogenicity	AI echogenicity	74	0.853	0.699–0.936	0.825	0.680–0.913	—	29	33	7	5
shape	AI shape	74	0.919	0.825–0.965	0.250	0.089–0.532	—	57	3	9	5
Margin	AI margin	74	0.662	0.540–0.765	0.889	0.565–0.980	—	43	8	1	22
echogenic foci	AI echogenic foci	74	0.842	0.624–0.945	0.750	0.623–0.845	—	16	42	14	3

Abbreviations: Se, sensitivity; Sp, specificity. Sensitivity and specificity CIs are Wilson 95% CIs. Operating point defined by Youden's J. TP, true positive; TN, true negative; FP, false positive; FN, false negative

Supplementary Table S2. Sensitivity analysis across ground-truth thresholds for AI size measurements

Sensitivity analysis assessing agreement and discrimination analysis *of AI-based size measurements across ground-truth (GT) thresholds of 5, 10, and 15 mm. For each axis and threshold, the table reports AUROC, the Youden-optimized AI decision threshold (mm), sensitivity, and specificity.*

Axis	GT_threshold_mm	AUROC	AI_threshold_Youden	Sensitivity	Specificity
Transverse	5	0.786	5.3	0.986	1.0
Transverse	10	0.613	10.5	0.87	1.0
Transverse	15	0.669	14.8	0.933	0.977
AP	5	0.23	4.7	0.938	0.9
AP	10	0.608	8.9	0.914	0.923
AP	15	0.661	13.4	0.889	0.982
Longitudinal	5	0.768	7.2	0.886	1.0
Longitudinal	10	0.797	11.4	0.923	0.955
Longitudinal	15	0.822	17.3	0.765	1.0
